# Seat Belt Aorta in a Paediatric Patient: Conservative Management with Eight Year Follow Up to Adulthood

**DOI:** 10.1016/j.ejvsvf.2025.12.005

**Published:** 2026-01-10

**Authors:** Alice Fourrier, Bahaa Nasr, Thomas Hebert, Benjamin Espinasse

**Affiliations:** a Department of Vascular Medicine, Brest University Hospital, Brest, France; b Department of Vascular Surgery, Brest University Hospital, Brest, France; c Brest University, Institut National de la Santé et de la Recherche Médicale, Institut Mines-Télécom Atlantique, Unité Mixte de Recherche 1011 Laboratoire de Traitement de l'Information Médicale, Brest, France; d Department of Radiology, Brest University Hospital, Brest, France; e Brest University, Institut National de la Santé et de la Recherche Médicale, Unité Mixte de Recherche 1304 Groupe d'Étude de la Thrombose de Bretagne Occidentale, Brest, France

**Keywords:** BAAI, Blunt abdominal aortic injury, Paediatric trauma, Seat belt aorta, Vascular surgery, Vascular medicine

## Abstract

**Introduction:**

Seat belt syndrome can cause blunt abdominal aortic injury (BAAI) in children. No specific guidelines exist for managing aortic injury in the paediatric population, presenting challenges due to ongoing somatic growth and vascular development.

**Report:**

A 10 year old girl sustained BAAI in a high kinetic motor vehicle collision (90 km/h). Initial computed tomography revealed a grade III infrarenal abdominal aortic injury (circumferential intimal dissection extended to the common iliac arteries). Focal dilatation measured 13mm at the aortic segment compared to 11mm proximally. Despite injury severity, the patient remained haemodynamically stable with adequate lower extremity perfusion. Conservative management with antihypertensive therapy and intensive duplex ultrasound surveillance was pursued. The eight year follow up showed an increase of the ectatic segment from 14 to 22 mm, stability of the left common iliac artery stenosis, and no target lesion intervention.

**Discussion:**

Conservative management may avoid intervention during somatic growth, circumventing complications of undersized devices, endoprosthesis migration, and peri-operative risks. Haemodynamically stable paediatric BAAI with contained injury may be managed conservatively with intensive surveillance. Multicentre case registries are needed to establish evidence based guidelines.

## Highlights

• Blunt aortic injury from seat belts affects paediatric patients.
• Conservative management succeeded through eight years of growth.
• Serial ultrasound provided radiation free surveillance to adulthood.
• Stable growth ratio suggests healing not aneurysmal degeneration.
• Paediatric specific treatment guidelines are needed.

## Introduction

Garrett and Braunstein^1^ firstly described seat belt syndrome in 1962, characterising a constellation of injuries from seat belt restraint mechanisms. This syndrome includes injuries to solid or hollow viscera, vertebral Chance fractures, marked ecchymosis (seat belt sign), and vascular injuries involving the aorta (seat belt aorta).^2^ Vascular injuries range from contusion to complete aortic rupture.^3^ Management options include conservative therapy, open surgical repair, or endovascular intervention.

Current literature lacks clear guidelines for managing blunt abdominal aortic injury (BAAI) in paediatric patients, who present unique considerations related to somatic growth, prolonged life expectancy, and durable treatment strategies. The authors present a paediatric case of BAAI managed conservatively and followed for eight years to adulthood. The patient provided informed consent.

## report

A 10 year old female passenger sustained injuries in a high kinetic motor vehicle collision (90 km/h, 56 mph) with frontal impact in July 2016. The patient was wearing a three point seat belt and remained conscious throughout but complained of abdominal pain. She was promptly transferred to the paediatric emergency department where whole body computed tomography (CT) imaging was performed given the high energy mechanism of injury. Initial CT scan revealed pulmonary parenchymal contusions, cervical whiplash injury, and minimal retroperitoneal haemorrhage associated with aortic trauma consisting of a grade III infrarenal abdominal aortic injury with circumferential intimal dissection extended to the common iliac arteries. Focal dilatation measured 13 mm at the aortic segment compared 11 mm proximaly.

**Figure 1:**
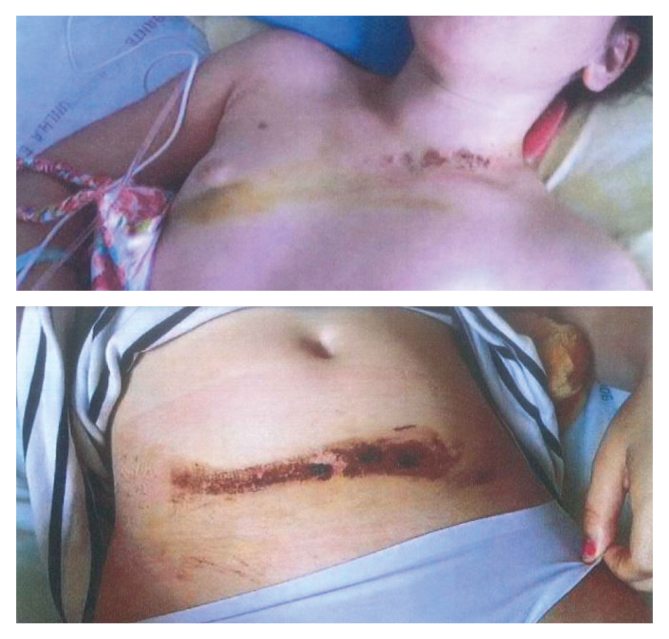
Photograph taken at the time of the accident. A seat belt sign is visible in the area where the body encountered the seat belt. Superficial dermabrasion predominates in the cervical and hypogastric regions, and skin ecchymosis predominates in the thoracic region.

The patient was transferred to the paediatric intensive care unit. On admission, she remained haemodynamically stable with a heart rate of 100 beats per minute, blood pressure of 122/65 mmHg without vasopressor support, oxygen saturation of 100%, and admission haemoglobin was 12.3 g/dL. The characteristic seat belt sign was present (Fig. 1). Despite diminished femoral pulses, the lower extremities were warm with capillary refill less than two seconds, and symmetrical dorsalis pedis and posterior tibial pulses were palpable bilaterally.

The multidisciplinary team opted for conservative management based on haemodynamic stability without vasopressor support, stable haemoglobin, contained aortic injury without free rupture or expanding haematoma, adequate distal perfusion despite diminished femoral pulses, and challenges of paediatric aortic intervention including undersized devices, risk of somatic growth related complications, and peri-operative risks.

**Figure 2:**
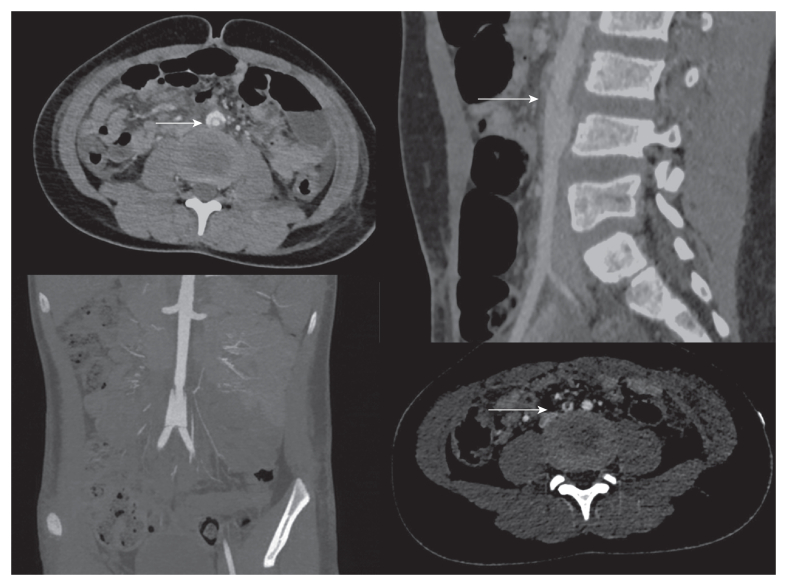
Abdominal computed tomography angiography performed 48 hours after the trauma. Top left: Axial slice showing circumferential intimal dissection of the infrarenal abdominal aorta. Top right: Sagittal slice showing floating intimal flap (arrow) with ectasia measuring 14 mm. Bottom left: Coronal slice showing intimal dissection. Bottom right: Axial slice showing intimal dissection at common iliac origins.

Follow up CT imaging at 48 hours using a dedicated vascular protocol (Fig. 2) revealed the persistence of a grade III BAAI (Azizzadeh classification), characterised by circumferential intimal disruption with mobile intimal flap, focal aneurysmal dilatation, and intimal flap extension to the common iliac artery. The injury represented a partial thickness aortic wall injury with circumferential intimal detachment involving the intima and media without complete transmural rupture through the adventitia. The dissection extended from below the inferior mesenteric artery ostium to the aortic bifurcation. The distal intimal flap was impacted at the common iliac artery origins, resulting in moderate stenosis bilaterally. The retroperitoneal haematoma had regressed, and the patient's haemoglobin remained stable.

Although the patient was normotensive on admission, antihypertensive therapy was initiated to minimise haemodynamic stress on the injured aortic wall, following principles adapted from acute aortic dissection management. Target systolic blood pressure was set at 100–110 mmHg. Intravenous labetalol and nicardipine were administered initially, subsequently simplified to labetalol monotherapy (5 mg/h).

Post-traumatic surveillance of the aortic injury was conducted using Duplex Ultrasound (DUS). The radiation free nature of ultrasound and its bedside availability made it the preferred modality for serial monitoring in this paediatric patient. The intimal dissection identified on CT imaging was well visualised by ultrasound. The mobile intimal flap was visualised within the aortic lumen, with the ectatic segment measuring 12 mm (a 2 mm difference compared with CT). Peak systolic velocity (PSV) at the injury site was 180 cm/s with a systolic velocity ratio (SVR) of 1.5. In the common iliac arteries, bilateral flow dampening was noted with PSVs of 220 cm/s on the right (SVR 2.1) and 240 cm/s on the left (SVR 2.3).

The patient was discharged after three weeks with stable aortic lesions on serial DUS. Physical activity was contraindicated, and blood pressure was controlled with labetalol (35 mg twice daily) targeting systolic pressure <110 mmHg.

**Figure 3:**
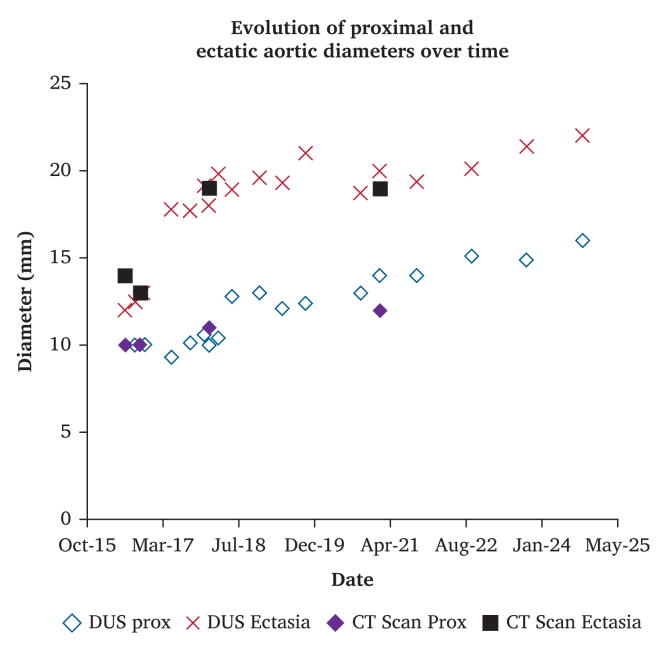
Evolution of proximal and ectatic aortic diameters over time, measured by Duplex Ultrasound (DUS) and computed tomography (CT).

Over eight years of surveillance from 2016 to 2024 combining DUS and CT angiography in 2018 and 2021 (Supplementary Videos S1 and S2), follow-up extended from age 10 to 18. Antihypertensive therapy was progressively reduced after eight months post-trauma, as systolic blood pressure remained below target levels and stable. During this period, the infrarenal abdominal aorta proximal to the injury increased from 10 to 16 mm, while the ectatic segment increased from 12 mm (initial DUS) to 22 mm (Fig. 3, Supplementary Table S1). An intimal flap remained visible within the proximal portion of the ectatic segment throughout follow up. The ectatic to proximal aorta diameter ratio remained relatively stable (range 1.3–1.9, Supplementary Fig. S2).

At the most recent follow up in 2024, the patient reported no lower extremity claudication, rest pain, or exercise intolerance. Physical examination revealed palpable femoral, popliteal, and pedal pulses bilaterally. Ankle brachial indices were 1.05 on the right and 0.95 on the left. The patient maintains an active lifestyle without vascular limitations. DUS evidence of chronic intimal dissection persisted in the proximal ectatic segment as mural thickening, without haemodynamically significant stenosis. Moderate stenosis of the left common iliac artery remained stable at 50–79% (PSV 360 cm/s, ratio 2.9) without impact on distal flow patterns, which remained triphasic (Supplementary Fig. S1), while right common iliac artery flow had normalised (PSV 120 cm/s, velocity ratio 1.1) compared with the initial flow dampening (PSV 220 cm/s, initial ratio 2.1).

Annual DUS examinations will continue while lesions remain stable. Cross sectional imaging (CT or magnetic resonance angiography) will be performed if DUS demonstrates progressive aneurysmal enlargement, increasing stenosis with haemodynamic symptoms, new concerning features or if intervention is considered. The patient was counselled regarding potential intervention should the lesion demonstrate concerning progression.

## Discussion

Literature regarding this condition remains limited, consisting primarily of case reports spanning several decades, providing insufficient evidence for optimal management strategies.^2^ The Western Trauma Association multicentre study by Shalhub *et al.*^4^ reported outcomes in adult BAAI, demonstrating that grade and haemodynamic status are key determinants of the management approach. However, paediatric specific data remain scarce.

This case report describes traumatic abdominal aortic injury secondary to a high kinetic motor vehicle accident with seat belt syndrome in a paediatric patient. Successful conservative management that avoided surgical or endovascular intervention during childhood was demonstrated, with the patient now having reached adulthood at final follow up with eight years of surveillance. The stability of the ectatic to proximal aorta diameter ratio is reassuring and suggests that the ectatic segment is enlarging proportionally with normal somatic aortic growth rather than demonstrating accelerated pathological aneurysmal degeneration, providing clinical evidence that continued conservative management remains appropriate.

This approach circumvented complications associated with paediatric aortic interventions, including risk of stenosis from undersized devices, endoprosthesis migration during somatic growth, and peri-operative anaesthetic and infection risks. Should future intervention become necessary, treatment would be less complex in the mature aortic anatomy.

The patient presented with a grade III injury. While adult literature indicates that grades III–IV typically require intervention, paediatric patients may justify conservative management in carefully selected cases with intensive monitoring and multidisciplinary expertise.

DUS was selected for surveillance based on the patient's young age (10 years), need for serial monitoring, and adherence to as low as reasonably achievable (ALARA) principles to minimise radiation exposure. While CT angiography would have provided optimal anatomic comparison with the 2016 baseline, DUS provided sufficient diagnostic information to monitor lesion stability, haemodynamic significance, and growth related changes.

Based on the authors’ experience, considerations for paediatric BAAI management are proposed. Haemodynamically stable patients with contained injuries (grades I–III), adequate distal perfusion, and no active haemorrhage may be candidates for non-operative management with intensive monitoring. Blood pressure should be maintained 20–30 mmHg below age adjusted norms to reduce wall stress. Intervention thresholds include rapid aneurysmal enlargement, haemodynamic instability, symptomatic limb ischaemia, or contained rupture. Long term planning should recognise that deferred intervention until skeletal maturity offers technical advantages with adult sized devices. A multidisciplinary approach is essential, incorporating paediatric intensivists, vascular surgeons, vascular physicians, and radiologists, with structured transition planning to adult care.

This case report has several limitations. Firstly, it represents a single patient's experience and cannot establish generalisable treatment algorithms. Secondly, there is a lack of a current CT angiogram for direct comparison with the initial 2016 CT scan. Thirdly, long term outcomes beyond eight years remain unknown. Additional research is needed to establish evidence based guidelines for clinical decision making in this patient population.

In conclusion, this case demonstrates that conservative management of grade III BAAI in a haemodynamically stable paediatric patient can result in a successful outcome without intervention through eight years of follow up. The patient transitioned from childhood to adulthood with preserved quality of life and vascular function. This case contributes to the limited literature on paediatric BAAI natural history but also highlights crucial knowledge gaps. Given the rarity of paediatric BAAI, multicentre case registries and collaborative reporting are needed to establish whether this conservative approach is generalisable and to develop consensus based management guidelines for this rare but challenging clinical scenario.

## Declaration of Generative AI and AI-assisted technologies in the writing process

During the preparation of this work, the authors used Claude (Anthropic) to enhance language clarity and readability. All content was subsequently reviewed and edited by the authors, who take full responsibility for the final manuscript.

## Appendix A Supplementary data

Multimedia component 1

Video 1 A CT 3d reconstruction of the aortic ectasia (Jan 2018, age 11).

Video 2 A CT 3d reconstruction of the aortic ectasia (February 2021, age 14).
