# Supplementary material for: Seat Belt Aorta in a Paediatric Patient: Conservative Management with Eight Year Follow Up to Adulthood
Source: EJVES Vasc Forum. 2026 Jan 10;65:83–6. doi: 10.1016/j.ejvsvf.2025.12.005 (PMC12969151; doi:10.1016/j.ejvsvf.2025.12.005)
Supplement: Multimedia component 1 [file mmc1.docx]

Supplementary Information

Table 1 : evolution of aortic proximal diameter, ectasia diameter and ratio ectasia/proximal aorta diameter.

| **Date/Year** | **Patient Age (years)** | **Imaging Modality** | **Aortic diameter proximal** | **Aortic Ectasia Diameter (mm)** | **Ratio** |
| --- | --- | --- | --- | --- | --- |
| **Jul-16** | **10** | **CT** | **10** | **14** | **1.4** |
| Jul-16 | 10 | DUS | 10 | 12 | **1.2** |
| **Aug-16** | **10** | **CT** | **10** | **13** | **1.3** |
| Sep-16 | 10 | DUS | 10 | 12.5 | **1.3** |
| Nov-16 | 10 | DUS | 10 | 13 | **1.3** |
| May-17 | 11 | DUS | 9.3 | 17.8 | **1.9** |
| Sep-17 | 11 | DUS | 10.1 | 17.7 | **1.8** |
| Dec-17 | 11 | DUS | 10.6 | 19.1 | **1.8** |
| **Jan-18** | **11** | **CT** | **11** | **19** | **1.7** |
| Jan-18 | 11 | DUS | 10 | 18 | **1.8** |
| Mar-18 | 12 | DUS | 10.4 | 19.8 | **1.9** |
| Jun-18 | 12 | DUS | 12.8 | 18.9 | **1.5** |
| Dec-18 | 12 | DUS | 13 | 19.6 | **1.5** |
| May-19 | 13 | DUS | 12.1 | 19.3 | **1.6** |
| Oct-19 | 13 | DUS | 12.4 | 21 | **1.7** |
| Oct-20 | 14 | DUS | 13 | 18.7 | **1.4** |
| **Feb-21** | **14** | **CT** | **12** | **19** | **1.6** |
| Feb-21 | 14 | DUS | 14 | 20 | **1.4** |
| Oct-21 | 15 | DUS | 14 | 19.4 | **1.4** |
| Oct-22 | 16 | DUS | 15.1 | 20.1 | **1.3** |
| Oct-23 | 17 | DUS | 14.9 | 21.4 | **1.4** |
| Oct-24 | 18 | DUS | 16 | 22 | **1.4** |

**
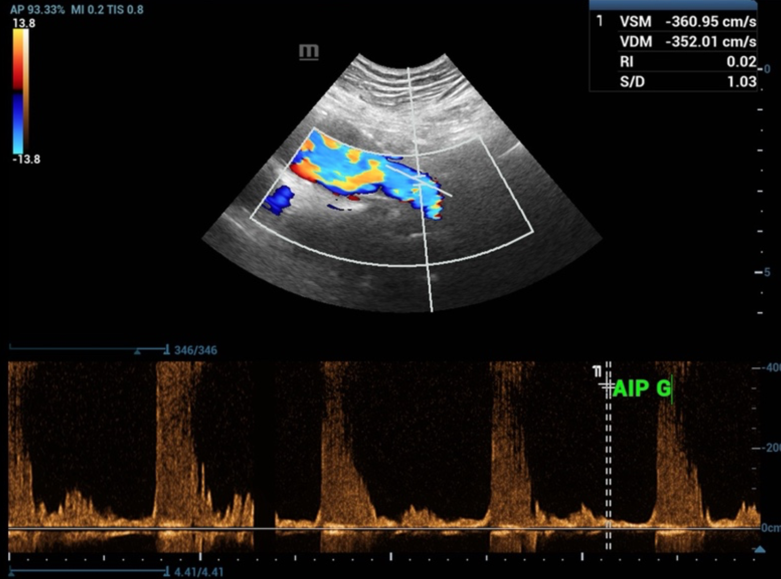

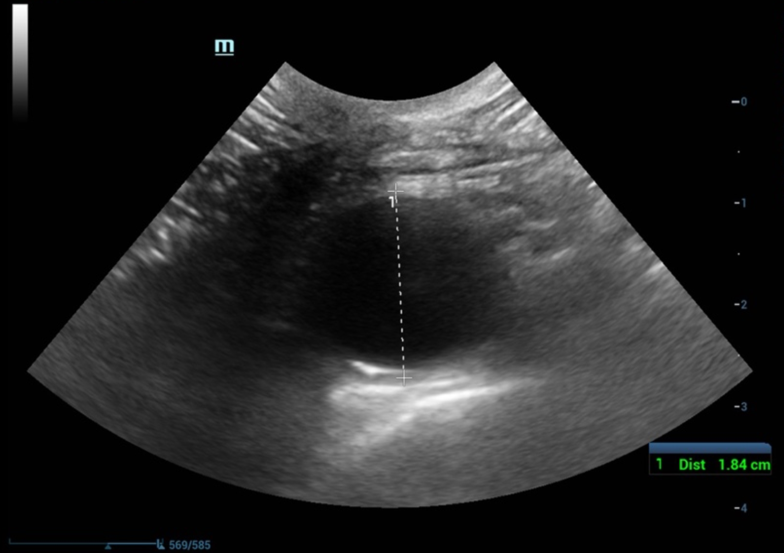

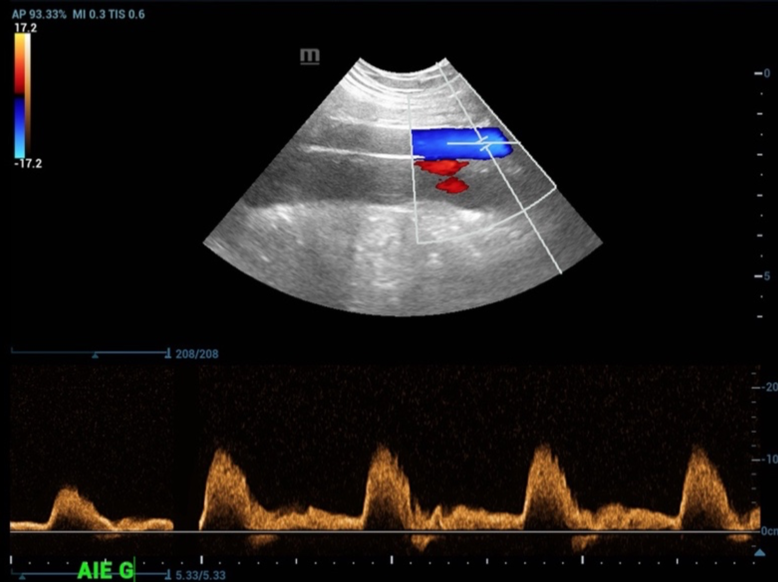

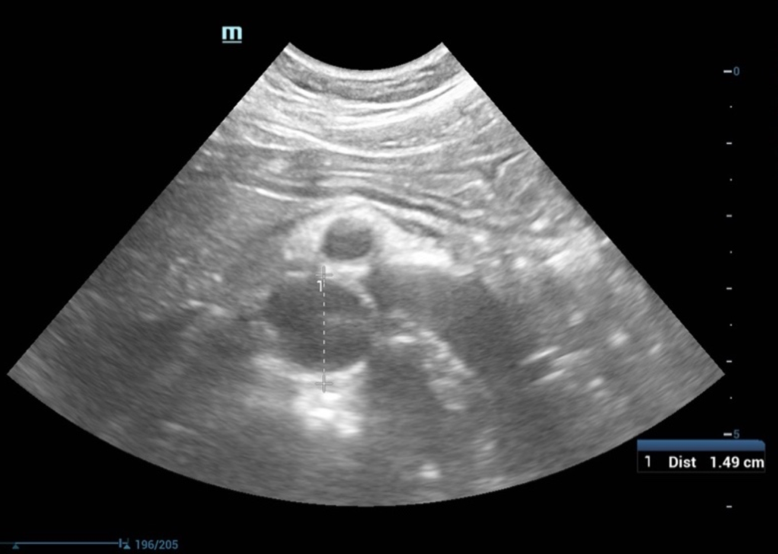
**Figure S1 : Doppler ultrasound performed 7 years post-injury (Age 17,). Top left: Proximal aorta measuring 15 mm. Top right: Ectatic segment measuring 18.1 mm. Bottom left: Left common iliac artery with moderate stenosis (PSV 360 cm/s). Bottom right: Left external iliac artery with non-dampened triphasic flow.

Figure S2 : Ratio Evolution of ectasia ovet aortic diameters with time, measured by DUS and CT.
